# Therapeutic applications of CRISPR-Cas9 gene editing

**DOI:** 10.3389/fgeed.2025.1724291

**Published:** 2025-12-16

**Authors:** Aditya Bharti, Joann Mudge

**Affiliations:** 1 Green Level High School, Cary, NC, United States; 2 National Center for Genome Resources, Santa Fe, NM, United States

**Keywords:** CRISPR-cas9, gene editing, therapeutics, clinical trials, cancer

## Abstract

CRISPR-Cas9 is a gene editing tool used extensively in biological research that is now making its way into clinical therapies. With the first CRISPR therapy obtaining approval by the United States’ Food and Drug Administration (FDA) in late 2023, we look at clinical trials of emerging therapies involving CRISPR-Cas9, currently the most prevalent CRISPR-based tool in these trials. A CRISPR-based therapy is currently approved for treatment of both sickle-cell anemia and transfusion-dependent β-thalassemia but clinical trials for CRISPR-based therapeutics include a much broader range of targets. CRISPR-Cas9 is being explored to treat cancer, infectious disease, and more. This review highlights CRISPR-Cas9 clinical trials registered at clinicaltrials.gov as of 12/31/2024.

## 1 Introduction

Precise gene editing has been made possible by co-opting an adaptive immune system first identified in bacteria. “Clustered Regularly Interspaced Short Palindromic Repeats” (“CRISPR”) genomic regions store bits of foreign DNA, allowing the organism to swiftly recognize and respond if these foreign invaders return. The Cas9 endonuclease, guided by RNA transcribed from the CRISPR array, finds and cleaves the foreign genetic material, removing the threat (Yoshizumi et al., 2018). This RNA-targeted endonuclease system enabled development of a precise, programmable gene editing tool that holds immense promise for treating intractable diseases (Abbott, 2016). Other CRISPR enzymes, such as Cas12a, are making their way into clinical trials. While Cas12a provides benefits over Cas9 in some situations, such as staggered doublestrand breaks that leaves overhangs leading to more consistent repair, and a motif recognition that works better in AT-rich sequence, it can also yield more off-target effects (Zetsche et al., 2015). Because the vast majority of CRISPR-based therapies currently in trials utilize the Cas9 enzyme, here we focus on CRISPR-Cas9-based therapies.

**FIGURE 1:**
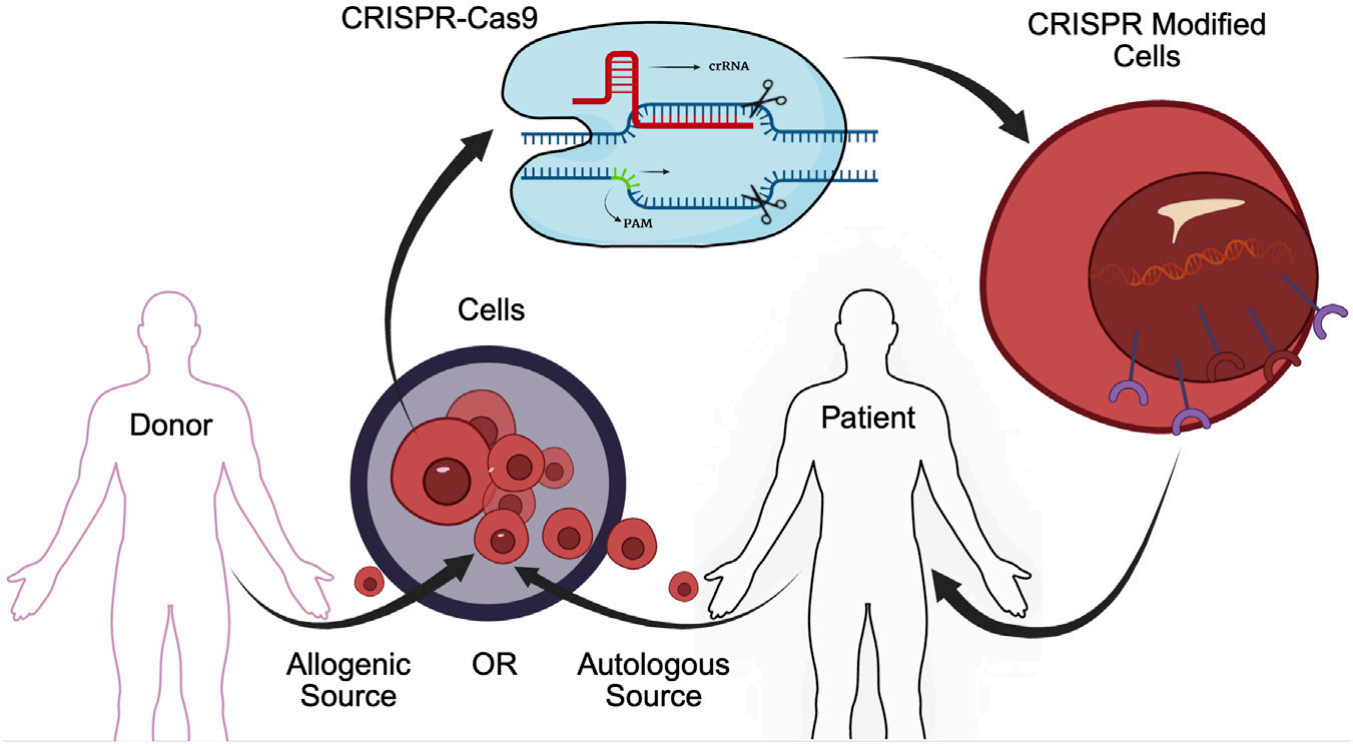
CRISPR-Cas9 therapies often involve removing cells from the patient’s body (autologous therapies) or obtaining cells from a donor (allogenic therapies), applying the therapy to alter the target gene, and (re)introducing the modified cells into the patient. The figure illustrates CAR-T therapy, often used in cancer but other CRISP-Cas9 therapies that edit cells outside of the body have a similar workflow.

Early CRISPR-Cas9 therapies targeted blood disorders, harvesting CD-34+ hematopoietic stem and progenitor cells (HSPCs) from patients (autologous) or donors (allogenic), modifying the cells using CRISPR-Cas9, then (re)introducing them into the patient (Figure 1). Blood cells derived from the modified HSPCs quickly become dominant as blood cells turn over. In addition to *ex-vivo* approaches that modify cells outside of a living organism, *in-vivo* CRISPR therapies can be injected directly into the patient. This review explores promising therapies described in complete and ongoing CRISPR-Cas9 clinical trials in any phase, registered at clinicaltrials.gov by 12/31/2024. (National Institutes of Health, 2022).

## 2 First approved CRISPR-Cas9 therapy

In late 2023, the first CRISPR-Cas9-based gene editing therapy (CASGEVY™) gained FDA approval for sickle cell disease (SCD) (FDA, 2023). SCD is caused by a β-globin (HBB) gene mutation, breaking the β-subunit of adult hemoglobin (HbA; 2 α- and 2 β-subunits). This leads to sickle-shaped red blood cells (RBCs), reduced blood flow, and less efficient oxygen delivery. The gene editing does not fix the HBB mutation, but rather increases fetal hemoglobin (HbF) expression (2 α- and 2 γ-subunits), avoiding the mutated β-subunit. HbF binds oxygen more strongly and is less likely to cause sickling. To increase HbF, CRISPR-Cas9 breaks the BCL11A gene, which normally represses HbF production. This therapy is more tolerable, effective, and permanent than transfusions and transplants (Singh et al., 2024).

More recently, CASGEVY was approved for transfusion-dependent β-thalassemia (TDT), an HBB mutation that results in insufficient β-globin. CASGEVY treats TDT with the same strategy as SCD, releasing the HbF block (Vertex Pharmaceuticals, 2024). Ongoing CASGEVY (CTX001) TDT/SCD trials measure engraftment stability, HbF levels, maintenance of transfusion independence, and mitigation of severe vaso-occlusive crises (NCT05356195,NCT03655678,NCT05477563,NCT03745287,NCT05329649,NCT04208529,NCT05951205).

## 3 Additional CRISPR-Cas9 therapies in clinic trials

Clinically-trialed CRISPR-Cas9 therapies target a broad range of diseases, bringing hope for treating intractable diseases (Table 1).

**TABLE 1:** CRISPR-Cas9 clinical trials.

Disease category	Intervention	Study numbers	Gene Target(s)	Therapeutic strategy/Delivery method (if *in vivo*)	Condition
Hemoglobinopathies	CTX001	**NCT05356195, NCT03655678, NCT05477563, NCT03745287, NCT05329649, NCT04208529, NCT05951205, NCT06287099**	BCL11A	*Ex vivo* gene disruption	Transfusion-dependent β -thalassemia (TDT)/Severe sickle cell disease (SCD)
	BRL-101	**NCT06287086, NCT06300723, NCT05577312**	BCL11A	*Ex vivo* gene disruption	Transfusion-dependent β -thalassemia (TDT)/Severe sickle cell disease (SCD)
	ET-01	NCT04925206	BCL11A	*Ex vivo* gene disruption	Transfusion-dependent β -thalassemia (TDT)
	Plerixafor + busulfan + gene-modified CD34^+^ cells	NCT06506461	BCL11A	*Ex vivo* gene disruption	Sickle cell disease (SCD)
	CRISPR-SCD001	NCT04774536	HBB	*Ex vivo* gene correction	Sickle cell disease (SCD)
	Nula-cel drug product	**NCT04819841**	HBB	*Ex vivo* gene correction	Sickle cell disease
	OTQ923	NCT04443907	HBG1 and HBG2	*Ex vivo* gene disruption	Sickle cell disease
Hematologic malignancies	CTX131	**NCT06492304**	CD70, TRAC, B2M, TGFBR2, Regnase-1	*Ex vivo* gene disruption + gene insertion	B-cell lymphoma/T-cell lymphoma/Acute myeloid leukemia (AML)
		**NCT04502446**	CD70, TRAC, MHC I ( β 2M)	*Ex vivo* gene insertion + gene knock-out	Relapsed/refractory T-cell lymphoma/Diffuse large B-Cell lymphoma
	CTX110	**NCT04035434**	TRAC, B2M	*Ex vivo* gene disruption + CAR insertion	Relapsed or refractory B-cell acute lymphoblastic leukemia/B-cell non-hodgkin lymphoma/B-cell lymphoma
	Universal dual specificity CD19 and CD20 or CD22 CAR-T cells	NCT03398967	TRAC	*Ex vivo* gene disruption + *ex* *vivo* CAR insertion	Relapsed or refractory B-cell leukemia/lymphoma
	CTX112	**NCT05643742**	TRAC, B2M, TGFBR2, Regnase-1 (ZC3H12A), CD70	*Ex vivo* gene disruption + site-specific CAR insertion	Relapsed or refractory B-cell malignancies
	UCART019	NCT03166878	TRAC, B2M	*Ex vivo* gene disruption	Relapsed or refractory CD19^+^ B-cell leukemia/lymphoma
	CT125A cells + cyclophosphamide + fludarabine	**NCT04767308**	CD5	*Ex vivo* gene disruption	Relapsed/refractory CD5^+^ hematopoietic malignancies
	PBLTT52CAR19	**NCT04557436**	TRAC, CD52	*Ex vivo* gene disruption	Relapsed/refractory B-cell acute lymphoblastic leukemia (B-ALL)
	Donor-derived CD34^+^ HSC with CRISPR/Cas9-mediated CD33 deletion + emtuzumab ozogamicin	**NCT05662904**	CD33	*Ex vivo* gene disruption	Relapsed/refractory acute myeloid leukemia (AML)
	REGV131 + LNP1265	**NCT06379789**	F9 (factor IX)	*In vivo* gene insertion/LNP (lipid nanoparticle)	Hemophilia B
	BE-101	**NCT06611436**	F9 (factor IX)	*Ex vivo* gene insertion	Hemophilia B
	NTLA-2002 + normal saline IV administration	**NCT06634420**	KLKB1	*In vivo* gene disruption/knockout/LNP	Hereditary angioedema (HAE)
	Biological NTLA-2002 + normal saline IV administration	**NCT05120830**	KLKB1	*In vivo* gene disruption/knockout/LNP	Hereditary angioedema (HAE)
	CTX120	NCT04244656	BCMA	*Ex vivo* gene disruption + insertion	Relapsed or refractory multiple myeloma
Solid tumor	Anti-mesothelin CAR-T cells	**NCT03545815**	PDCD1, TCR	*Ex vivo* gene disruption/knockout	Mesothelin-positive multiple solid tumors
	Mesothelin-directed CAR-T cells	**NCT03747965**	PDCD1	*Ex vivo* gene disruption/knockout	Mesothelin-positive multiple solid tumors
	TGF β R-KO CAR-EGFR T Cells	**NCT04976218**	TGFBR2	*Ex vivo* gene disruption/knockout	Advanced EGFR-positive solid tumors
	MT027 cells suspension	NCT06726564	B7-H3	*Ex vivo* gene insertion	Pleural malignant tumors
	Autologous CD19-star-t cell + fludarabine + cyclophosphamide	**NCT05631912**	TRAC, CD19	*Ex vivo* gene disruption + gene insertion	Relapsed/refractory B-cell non-hodgkin lymphoma (B-NHL)
	Allogeneic CD19-STAR T cell + fludarabine + cyclophosphamide	**NCT06321289**	TRAC, HLA-A/HLA-b, CIITA, PD-1	*Ex vivo* gene knockout (disruption) + gene insertion	Relapsed/refractory B-cell non-hodgkin lymphoma (B-NHL)
	ATHENA CAR-T (TRAC and Power3 genes KO) + fludarabine + cyclophosphamide	NCT06014073	TRAC, Power3 (SPPL3)	*Ex vivo* gene disruption (knock-out)	Relapsed/refractory B-cell non-hodgkin lymphoma (NHL)
	CTX131	NCT05795595	CD70 (the CAR target), TRAC, B2M, TGFBR2, Regnase-1	*Ex vivo* gene disruption + CAR insertion	Relapsed or refractory solid tumors
	Fludarabine + cyclophosphamide + CISH inactivated TIL + Aldesleukin + Pembrolizumab	**NCT05566223**	CISH	*Ex vivo* gene knockout	Metastatic non-small cell lung cancer (NSCLC)
	PD-1 KO T cells via TACE | transcatheter arterial chemoembolization	NCT04417764	PDCD1	*Ex vivo* gene disruption/knockout	Advanced hepatocellular carcinoma
	Fludarabine + cyclophosphamide + Interleukin-2	NCT03044743	PDCD1	*Ex vivo* gene disruption/knockout	Advanced-stage epstein–Barr virus (EBV)–associated malignancies
	Cyclophosphamide + PD-1 knockout T Cells	NCT02793856	PDCD1	*Ex vivo* gene disruption/knockout	Metastatic/advanced non-small cell lung cancer (NSCLC)
	MT027 cells suspension	NCT06742593, NCT06737146	CD276/B7-H3	*Ex vivo* gene insertion	Recurrent or progressive high-grade glioma
	Cyclophosphamide + fludarabine + tumor-infiltrating lymphocytes (TIL) + Aldesleukin	NCT04426669	CISH	*Ex vivo* gene knockout	Metastatic gastrointestinal epithelial cancer
	PD-1 knockout T Cells	NCT03081715	PDCD1	*Ex vivo* gene knockout/disruption	Esophageal cancer
	AJMUC1–PD-1 KO anti-MUC1 CAR-T cells	**NCT05812326**	PD-1	*Ex vivo* gene knockout/disruption	MUC1-positive advanced breast cancer
Infectious disease	CAZ/AVI + Aztreonam + conventional treatment	**NCT05850871**	Drug resistance/virulence genes	*In vivo* CRISPR-Cas9/(delivery method not specified)	Carbapenem-resistant enterobacteriaceae infection
	CCR5 gene Modification	**NCT03164135**	CCR5	*Ex vivo* gene disruption/knockout	HIV-1 infection
	EBT-101	**NCT05144386**	HIV proviral DNA	*In vivo* gene excision/disruption/AAV9 viral vector (IV)	HIV-1 infection
	TALEN + CRISPR/Cas9 therapy	**NCT03057912**	HPV E6 + E7 oncogenes	*Ex vivo* gene disruption/knockout	HPV-related cervical intraepithelial neoplasia I/malignant neoplasm
	PD-1 and ACE2 knockout T Cells (triple infusion)	**NCT04990557**	PD-1, ACE2	*Ex vivo* gene knockout/disruption	COVID-19 (SARS-CoV-2 infection)
Ophthalmic disorders	BD113vVLP	**NCT06465537**	MYOC	*In vivo* gene disruption/knockout/Virus-like particle (VLP)	Primary open-angle glaucoma (POAG)
	BD111 adult single Group dose	**NCT04560790**	UL8, UL29	*In vivo* gene disruption/mRNA CRISPR/Cas9 injected via corneal injection (intra-corneal)	Refractory viral keratitis
	ZVS203e	**NCT05805007**	RHO	*In vivo* gene inactivation/knockout/AAV	Retinitis pigmentosa
	EDIT-101	**NCT03872479**	CEP290	*In vivo* gene correction/AAV5	Leber congenital amaurosis type 10 (LCA10)
Other conditions	VCTX211	NCT05565248	B2M, TXNIP, PD-L1, HLA-E, TNFAIP3, and MANF	*Ex vivo* gene disruption + insertion	Type 1 diabetes (T1D
	VCTX210A unit	NCT05210530	B2M, TXNIP, PD-L1, HLA-e	*Ex vivo* gene disruption + insertion	Type 1 diabetes Mellitus (T1D)
	NTLA-2001	**NCT04601051**	TTR	*In vivo* gene disruption/knockout/LNP	Transthyretin-related (ATTR) familial amyloid polyneuropathy/Wild-type transthyretin cardiac amyloidosis

All study numbers in bold are outlined within the review.

### 3.1 Hemoglobinopathies

Further clinical trials are underway for hemoglobinopathies (including SCD and TDT), diseases reduced hemoglobin levels that compromise oxygen delivery. BRL-101s treatment of SCD/TDT also targets the BCL11A gene, disrupting BCL11A’s enhancer, thereby lowering transcription and increasing HbF production. With a safety profile similar to that of the required autologous transplantation, BRL-101 enables transfusion independence and increased HBF and HBA levels (NCT06287099,NCT06287086,NCT06300723,NCT05577312) (Fu et al., 2023; Fu et al., 2022). Other hemoglobinopathy therapies directly target the HBB mutation. GPH101 edits HSPCs to reverse HBB’s valine to glutamic acid change in β-thalassemia (NCT04819841) (Kanter et al., 2021).

### 3.2 Cancer

CRISPR-Cas9 therapies combating cancers often use Chimeric Antigen Receptor T cell (CAR-T) therapy, a type of immunotherapy increasingly employing CRISPR’s precision. In CAR-T therapy, patient (autologous) or donor (allogenic) T cells are edited *ex-vivo* to recognize and kill cancer cells. A synthetic gene is inserted that encodes a chimeric antigen receptor (CAR) containing an antigen binding domain targeting cancer cell surface proteins. In leukemias, lymphomas and myelomas, these cancer cell surface antigens include various Cluster of Differentiation (CD) genes, which create important functional proteins on the surface of white blood cells (Zhang et al., 2017).

In multiple myeloma, a B cell-derived cancer, the over-expressed B-cell Maturation Antigen (BCMA), essential for B cell maturation, survival, and proliferation, is targetted (NCT04244656) (Rinaldi et al., 2022). The mutation or overexpression of Epidermal Growth Factor Receptor (EFGR) is often seen in cancers, increasing uncontrolled cell growth, making it another important antigen target in some cancers (NCT04976218) (Sasaki et al., 2013). An important target for mesotheliomas, cancers derived from the lining of different organs, is the mesothelin gene. This is an especially attractive target because this gene’s expression is limited to mesothelial cells, a cell type that is dispensible (NCT03545815,NCT03747965,NCT05812326) (Hassan et al., 2016).

Manipulating additional genes beyond the CAR insertion has improved CAR-T therapy’s effectiveness and longevity. Most disrupt the T cell receptor α chain (TRAC) gene by inserting the CAR into it, which also ensures uniform CAR expression. Without the α-subunit, the T cell receptor (TCR) is not functional. This increases therapy effectiveness by reducing spontaneous activation and differentiation of the modified T cells, avoiding T cell exhaustion. In addition, the lack of the TCR protein, which normally recognizes foreign material, helps avoid graft vs. host disease (GvHD), opening up CAR-T therapy to allogenic sources, reducing costs and timelines, and standardizing treatment (Eyquem et al., 2017; Lonez and Breman, 2024; Terrett et al., 2023).

CRISPR Therapeutics’s allogenic CAR-T therapies are being improved by editing additional genes beyond the CAR insertion and its disruption of TRAC. CTX110 (NCT04035434), targeting CD19^+^ cancers (B cell leukemias and lymphomas), and CTX130 (NCT04502446 and NCT04438083), targeting CD70^+^ cancers (T cell lymphomas and renal cell carcinomas), both knockout the β2M gene, a subunit of the major histocompatibility complex class 1 (MHC-I) subunit. The broken MHC-I protein prevents donor CAR-T cells from being recognized and destroyed by the patient’s immune system (host vs. graft disease (HvGD) (Terrett et al., 2023; McGuirk et al., 2022).

The next-generation drug versions, CTX112 for CD19^+^ cancers (NCT05643742) and CTX131 (NCT06492304) for CD70^+^ cancers, improve on their counterparts through additional gene knockouts. Regnase-1 normally tamps down on cytokine secretion and, by extension, the immune system. The Regnase-1 knockout, therefore, keeps the immune response strong. Likewise, Transforming Growth Factor-beta (TGF-β) receptor type 2 (TFGBR2) knockouts create a CAR-T cell without a receptor to recognize the (TGF-β) produced in the tumor microenvironment that would normally inhibit the T cell. In CTX131, CD70 is also knocked out, preventing fratricide in CD70-targeting CAR-T cells. The improvements are stark. For example, CTX112 is 10X more potent than CTX110 with improvements in persistence and anti-tumor effects (Kalaitzidis et al., 2023).

Further genes been identified whose disruption in CAR-T cells can lead to therapeutic improvements (Moradi et al., 2024; Feng et al., 2024). Knockout of the Programmed Cell Death Protein 1 (PDCD-1) gene can help keep anti-tumor activity strong and avoid immune suppression and T cell exhaustion. PDCD-1 creates the PD-1 protein. When PD-1 binds its ligand (PD-L1), it acts as a brake, inactivating T cells. Tumors take advantage by overexpressing PD-L1, allowing them to inactivate immune cell that recognized the cancer, thereby evading the antitumor immune response and leading to T cell exhaustion (Munari et al., 2021; Moradi et al., 2024). Several clinical trials (NCT03545815, NCT03747965, NCT05812326) deploy PDCD-1 knockout CAR-T cells against mesothelin + breast and other solid tumors. In one case (NCT03747965), the GC008t therapy stabilized disease in four patients and achieved tumor shrinkage for two patients, though engraftment could be improved (Wang et al., 2020).

One autologous clinical trial (NCT05566223) uses CRISPR-Cas9 to knockout the CISH (Cytokine-induced SH2 protein) gene in tumor infiltrating lymphocytes (TILs), a type of T cell that penetrates solid tumors. CISH limits T cell activation and signaling, so its disruption keeps anti-tumor responses high. This therapy treats non-small cell lung cancer (NSCLC), which accounts for ∼ 85% of diagnosed lung cancers, with lung cancers being the leading cause of cancer-related deaths globally (Gridelli et al., 2015).

CRISPR-Cas9 is also used to alter CAR-T cells to cope with concurrent monoclonal antibody treatment. One autologous therapy (NCT05662904) treats acute lymphoblastic leukemia (ALL) by inactivating the CD33 gene in the patient’s HSPCs to make them immune to the CD33-specific antibody-drug conjugate Gemtuzumab-ozogamicin (GO), allowing escalation of GO doses (Godwin et al., 2017). In another study, PBLTT52CAR19 targets CD19^+^ pediatric B cell ALL (NCT04557436). The disruption of the CD52 gene allowed the concurrent use of Alemtuzumab (Drugs.com, 2024), an anti-CD52 monoclonal therapy. Four of six patients showed CAR-T cell proliferation, achieved remission, and then received allogenic stem cell transplantation for a more permanent therapy (Ottaviano et al., 2022).

CRISPR-cas9 editing can also introduce safety switches into cancer therapies to avoid serious side effects, including Cytokinin Release Syndrome (CRS) and immune cell-associated neurotoxicity syndrome (ICANS) (Xiao et al., 2021). CT125A is an autologous CAR-T cell therapy that targets CD5^+^ hematologic malignancies, including T cell-derived leukemias and lymphomas (NCT04767308). The endogenous CD5 gene was disrupted using CRISPR-Cas9 to avoid fratricide. A safety switch was added to the CAR-T cells by editing a truncated epidermal growth factor receptor (tEGFR) into the genome. The resulting receptor, though not functional, was still recognized by Cetuximab, a monoclonal antibody therapy, killing the CAR-T cells when administered to the patient. Clinical outcomes were mixed. One patient went into complete remission but died of sepsis and multi-organ dysfunction. The other two patients achieved partial remission but one relapsed. As expected, the therapy caused CRS, but cetuximab administration eliminated most (but not all) CAR-T cells, limiting toxicity. Nevertheless, this study showed that safety switches can be viable strategies for limiting patient exposure to therapies with dangerous side effects (Lin et al., 2024).

Improvements over CARs are being tested, including STAR (Synthetic TCR and Antigen Receptor) T cell therapy. STAR-T therapy uses a construct that mimics TCRs, increasing sensitivity to the cancer-presented antigens, which is especially important in solid tumors with low antigen density (Huang et al., 2024). Two related studies (NCT05631912:autologous and NCT06321289: allogenic) are trialing CD19-targeting STAR-T therapy for B cell non-Hodgkin’s lymphoma. Additional knockouts of TRAC, PDCD-1, human leukocyte antigen (HLA)-A/B, and Class II Transactivator (CIITA) strengthened the intervention. In addition to reducing the immune suppression, delaying T cell exhaustion, and increasing anti-tumor activity with the TRAC and PDCD-1 knockouts, knockouts of HLA-A/B and CIITA, which are subunits of MHCI and MHCII proteins, respectively, reduce the recognition of allogenic STAR-T cells as foreign, thereby reducing the risk of GvHD.

These are some of many promising CRISPR-Cas9-based cancer therapies and strategies. The number of antigen targets is expanding, additional constructs are improving on CARs, and therapies are becoming more sophisticated with additional gene edits to improve longevity and safety and keep immune and anti-tumor functions high.

### 3.3 Infectious disease

CRISPR-Cas9 therapies can also fight infectious disease, either by targeting host or pathogen genes. Two clinical trials explore unique methods to treat Acquired Immunodeficiency Syndrome (AIDs), caused by human immunodeficiency virus I (HIV-1). These therapies target the host CC chemokine receptor 5 (CCR5) gene, which is one of the co-receptors that HIV-1 uses to enter the host’s CD4^+^ lymphocytes, thereby destroying a critical part of the host’s immune function. A frameshifting 32-nt deletion in CCR5 occurs naturally in a small proportion of the human population. This CCR5-\upDelta 32 mutation, when homozygous, prevents HIV-1 from entering the cell, allowing infected individuals (“HIV controllers”) to live with the virus (Oppermann, 2004; Carrington et al., 1997).

One allogenic study (NCT03164135) used CRISPR-Cas9 to modify donor HSPCs, ablating the CCR5 receptor to make the immune cells resistant to HIV-1. This study was designed for HIV patients who also had a hematologic malignancy that required stem cell transplantation, creating an opportunity to simultaneously test CCR5 ablation with minimal additional risk to the patient. One HIV-positive patient in this study had ALL. Transplantation and long-term engraftment was achieved. However, CCR5 was disrupted in only ∼ 5% of lymphocytes (Xu et al., 2019).

Another AIDS therapy, EBT-101, uses CRISPR-Cas9 to disrupt the HIV-1 genome in aviremic patients (patients with latent infections and no detectable blood virus levels (NCT05144386). Initial results met safety benchmarks and temporarily suppressed viral reservoirs (Johnson, 2024).

Persistent human papillomavirus (HPV) infection, the major cause of cervical cancer, is also being targeted by CRISPR-Cas9 therapies. The viral E6 and E7 oncoproteins inactivate host tumor suppressor genes promoting uncontrolled cell growth (Narisawa-Saito and Kiyono, 2007). Although small interfering RNA targeting of these oncogenes may temporarily inhibit HPV, it does not destroy the viral genes (Hu et al., 2015). Gene editing by administration of a CRISPR-Cas9 E6/E7-targeting plasmid in a gel reduced E6/E7 DNA and expression, initiated cell death, and prevented tumor growth (NCT03057912) (Hu et al., 2014).

The SARS-CoV-2 virus, which causes COVID-19, is targeted in a study that uses CRISPR-Cas9 to ablate the host PDCD1 and ACE2 receptor genes in CD8^+^ virus-reactive memory T cells (NCT04990557). PDCD-1 was knocked out because its upregulation during COVID-19 infection, even in patients with mild symptoms, promotes T-cell exhaustion. Knocking out the ACE2 receptor removes SARS-CoV-2’s main entry path into the modified T cell (Scialo et al., 2020).

CRISPR-Cas9 therapies are also beginning to target bacterial pathogens. One study uses CRISPR-Cas9 to disrupt virulence and β-lactam antibiotic resistance genes in Enterobacteriaceae genomes (NCT05850871). Because antibiotic resistance genes are horizontally transferred between species, including pathogens that cause different diseases, individual therapies could potentially target multiple pathogens and diseases.

### 3.4 Ophthalmic disorders

CRISPR-Cas9 therapies work well for eye diseases because they can be injected directly into the relevant eye tissue. In Intraocular Hypertensive Primary Open Angle Glaucoma (POAC), increased intraocular pressure damages the optic nerve, leading to blindness (Quigley et al., 1983). Dominant mutations in the cytosketetal myocilin (MYOC) gene, which is expressed in the trabecular meshwork where intraocular pressure is regulated, can cause POAC. The BD113 therapy is delivered in a virus-like particle (VLP) by eye injection to knockdown or knockout the mutated MYOC gene, reducing the amount of mutated protein (NCT06465537).

Another VLP therapy (BD111) is injected into the cornea to treat recalcitrant herpes stromal keratitis, which can cause infectious blindness (NCT04560790). The therapy uses CRISPR-Cas9 to disrupt the herpes simplex virus type 1 (HSV-1) genome. No HSV-1 was detected in follow-ups, averaging 18 months (Wei et al., 2023).

Reinitis pigmentosa results in rod cell loss, leading to night blindness, and the gradual loss of cone cells, leading to tunnel vision or blindness. The therapy (ZVS203e) is administered by subretinal injection and fixes a causal rhodopsin (RHO) gene mutation to create a functional protein that is activated under low light conditions (NCT05805007) (Nathans and Hogness, 1984; National Library of Medicine, 2025).

Another CRISPR-Cas9 therapy (EDIT-101) targets Leber Congenital Amaurosis 10 (LCA10) (NCT03872479). A homozygous mutation in the centrosomal protein 290 (CEP290) gene causes retinal degeneration leading to blindness or severe vision loss at birth or shortly thereafter (den Hollander et al., 2006). The mutation causes an additional splice site that forms a cryptic (additional) exon. Initial clinical trial results established safety and 75% of participants showed improved vision.

### 3.5 Other conditions

Hemophilia B is a bleeding disorder caused by a mutated coagulation Factor IX (FIX) gene that results in insufficient FIX (Kurachi and Kurachi, 2000). CRISPR-Cas9-based therapies insert wildtype FIX gene into liver and B cells, enabling clotting factor production (NCT06379789,NCT06611436).

Hereditary Angioedema (HAE) results in debilitating or fatal swelling under the skin. Treatments target kallikrein, a protease encoded by the KLB1 gene, which causes swelling when overproduced in blood plasma (Longhurst et al., 2022; Banerji et al., 2017). NTLA-2002 is a CRISPR-Cas9-based therapy that disrupts KLB1 in liver cells, reducing plasma kallikrein levels (Longhurst et al., 2022). Initial results established safety and showed a reduction in plasma kallikrein levels (NCT05120830,NCT06634420).

Transthyretin amyloidosis is a disease resulting from accumulation of misfolded proteins from a mutated or wildtype transthyretin (TTR) gene, into harmful amyloid fibril deposits, leading to polyneuropathy and/or cardiomyopathy Ruberg and Berk (2012). NTLA-2001 (NCT04601051), delivered *in vivo* through lipid nanoparticles, showing that systemic *in vivo* gene editing is possible (Gillmore et al., 2021).

## 4 Discussion

The recent CASGEVY FDA approval and the number of CRISPR-Cas9-based therapies in clinical trials, promise transformative therapies on the horizon. Personalized CRISPR-based therapies are also emerging. In May 2025, a personalized CRISPR-Cas9-based therapy was developed to treat an infant whose carbamoyl-phosphate synthetase 1 (CPS1) gene was mutated, thereby preventing the breakdown of byproducts of protein metabolism in the liver, leading to ammonia toxicity. The therapy, delivered in two doses via lipid nanoparticles, corrected the mutation, allowing the patient to tolerate high dietary protein, even while halving his nitrogen-scavenger drug dose, with no severe adverse effects (Musunuru et al., 2025).

But hurdles to CRISPR-Cas9-based therapies persist. Some therapies suffer from low engraftment or editing rates, do not work or have serious or even fatal side effects in some patients, or are subject to relapse (Wang et al., 2020; Xu et al., 2019; Xiao et al., 2021; Hamilton et al., 2024; Ozdemirli et al., 2024). Delivery methods need improvement. Early therapies focused on diseases that could be treated through *ex-vivo* therapies. Then *in-vivo* therapies that can be injected directly into the affected tissue were created but they suffer from lower delivery efficiency, off-target effects, and instability (Rostami et al., 2024). In addition, CRISPR-Cas9-based therapies are expensive, not widely accessible, and threaten to increase healthcare inequities. With CASGEVY originally priced at $2.2M/patient and the customized CPS1 treatment costing $2M/dose, urgent calls to reduces costs have been made (Rueda et al., 2024; Witkowsky et al., 2023; Ledford, 2025).

Nevertheless, steady progress is being made in CRISPR-Cas9-based therapies. Editing additional genes, such as building in safety switches in CAR-T therapy, improves both efficacy and safety (Xiao et al., 2021; Eyquem et al., 2017; Lonez and Breman, 2024; Terrett et al., 2023). Delivery methods are being explored that shift therapies from systemic to local treatments, increasing efficacy and lowering risk (Rostami et al., 2024). Cost-reduction solutions have been identified, including structural changes to healthcare institutions, changes in manufacturing and licensing, increased public investment, and the development of edited “off-the-shelf” donor cells and modular therapies that can be easily altered for different diseases (Rueda et al., 2024; Witkowsky et al., 2023). These trajectories indicate that emerging CRISPR-based therapies will provide improved opportunities for safely and effectively managing or even curing currently intractable diseases.

## References

[B1] Abbott A. (2016). A crispr vision. Nature 532, 432–434. 10.1038/532432a 27121823

[B2] Banerji A. Busse P. Shennak M. Lumry W. Davis-Lorton M. Wedner H. J. (2017). Inhibiting plasma kallikrein for hereditary angioedema prophylaxis. N. Engl. Journal Medicine 376, 717–728. 10.1056/NEJMoa1605767 28225674

[B3] Carrington M. Kissner T. Gerrard B. Ivanov S. O’Brien S. J. Dean M. (1997). Novel alleles of the chemokine-receptor gene ccr5. Am. J. Hum. Genet. 61, 1261–1267. 10.1086/301645 9399903 PMC1716101

[B5] den Hollander A. I. Koenekoop R. K. Yzer S. Lopez I. Arends M. L. Voesenek K. E. (2006). Mutations in the cep290 (nphp6) gene are a frequent cause of leber congenital amaurosis. Am. J. Hum. Genet. 79, 556–561. 10.1086/507318 16909394 PMC1559533

[B6] Drugs.com (2024). Alemtuzumab (multiple sclerosis) (monograph). Available online at: https://www.drugs.com/monograph/alemtuzumab-multiple-sclerosis.html.

[B7] Eyquem J. Mansilla-Soto J. Giavridis T. Van Der Stegen S. J. Hamieh M. Cunanan K. M. (2017). Targeting a car to the trac locus with crispr/cas9 enhances tumour rejection. Nature 543, 113–117. 10.1038/nature21405 28225754 PMC5558614

[B8] FDA (2023). FDA approves first gene therapies to treat patients with sickle cell disease. Available online at: https://www.fda.gov/news-events/press-announcements/fda-approves-first-gene-therapies-treat-patients-sickle-cell-disease.

[B9] Feng X. Li Z. Liu Y. Chen D. Zhou Z. (2024). Crispr/cas9 technology for advancements in cancer immunotherapy: from uncovering regulatory mechanisms to therapeutic applications. Exp. Hematology and Oncology 13, 102. 10.1186/s40164-024-00570-y 39427211 PMC11490091

[B10] Fu B. Liao J. Chen S. Li W. Wang Q. Hu J. (2022). Crispr–cas9-mediated gene editing of the bcl11a enhancer for pediatric *β*0/*β*0 transfusion-dependent *β*-thalassemia. Nat. Medicine 28, 1573–1580. 10.1038/s41591-022-01906-z 35922667

[B11] Fu B. Zhang X. Wang L. Liao J. Chen S. Zheng B. (2023). S271: an updated follow-up of brl-101, crispr-cas9-mediated gene editing of the bcl11a enhancer for transfusion-dependent beta-thalasse. HemaSphere 7, e406095b. 10.1097/01.hs9.0000967996.40609.5b 35922667

[B12] Gillmore J. D. Gane E. Taubel J. Kao J. Fontana M. Maitland M. L. (2021). Crispr-cas9 *in vivo* gene editing for transthyretin amyloidosis. N. Engl. J. Med. 385, 493–502. 10.1056/NEJMoa2107454 34215024

[B13] Godwin C. Gale R. Walter R. (2017). Gemtuzumab ozogamicin in acute myeloid leukemia. Leukemia 31, 1855–1868. 10.1038/leu.2017.187 28607471 PMC13345506

[B14] Gridelli C. Rossi A. Carbone D. P. Guarize J. Karachaliou N. Mok T. (2015). Non-small-cell lung cancer. Nat. Reviews Dis. Primers 1, 1–16. 10.1038/nrdp.2015.9 27188576

[B15] Hamilton M. P. Sugio T. Noordenbos T. Shi S. Bulterys P. L. Liu C. L. (2024). Risk of second tumors and t-cell lymphoma after car t-cell therapy. N. Engl. J. Med. 390, 2047–2060. 10.1056/NEJMoa2401361 38865660 PMC11338600

[B16] Hassan R. Thomas A. Alewine C. Le D. T. Jaffee E. M. Pastan I. (2016). Mesothelin immunotherapy for cancer: ready for prime time? J. Clin. Oncol. 34, 4171–4179. 10.1200/JCO.2016.68.3672 27863199 PMC5477819

[B17] Hu Z. Yu L. Zhu D. Ding W. Wang X. Zhang C. (2014). Disruption of HPV16-E7 by CRISPR/Cas system induces apoptosis and growth inhibition in HPV16 positive human cervical cancer cells. Biomed. Res. Int. 2014, 612823. 10.1155/2014/612823 25136604 PMC4127252

[B18] Hu Z. Ding W. Zhu D. Yu L. Jiang X. Wang X. (2015). Talen-mediated targeting of hpv oncogenes ameliorates hpv-related cervical malignancy. J. Clinical Investigation 125, 425–436. 10.1172/JCI78206 25500889 PMC4382249

[B19] Huang D. Li Y. Rui W. Sun K. Zhou Z. Lv X. (2024). Tcr-mimicking star conveys superior sensitivity over car in targeting tumors with low-density neoantigens. Cell Rep. 43, 114949. 10.1016/j.celrep.2024.114949 39520682

[B20] Johnson V. (2024). Crispr-editing ebt-101 therapy safe, temporarily suppresses hiv infection

[B21] Kalaitzidis D. Ghonime M. Chain R. Jaishankar N. Settipane D. Padalia Z. (2023). 274 development of ctx112 a next generation allogeneic multiplexed crispr-edited cart cell therapy with disruptions of the tgfbr2 and regnase-1 genes for improved manufacturing and potency. J. Immunother. Cancer, A314. 10.1136/jitc-2023-SITC2023.0274

[B22] Kanter J. DiPersio J. F. Leavey P. Shyr D. C. Thompson A. A. Porteus M. H. (2021). Cedar trial in progress: a first in human, phase 1/2 study of the correction of a single nucleotide mutation in autologous hscs (gph101) to convert hbs to hba for treating severe scd. Blood 138, 1864. 10.1182/blood-2021-152892

[B23] Kurachi K. Kurachi S. (2000). Genetic mechanisms of age regulation of blood coagulation: factor ix model. Arteriosclerosis, Thrombosis, Vascular Biology 20, 902–906. 10.1161/01.atv.20.4.902 10764652

[B24] Ledford H. (2025). Ultra-powerful crispr treatment trialled in a person for first time. Nature 641, 1083. 10.1038/d41586-025-01593-z 40389531

[B25] Lin H. Cheng J. Zhu L. Zeng Y. Dai Z. Zhang Y. (2024). Anti-cd5 car-t cells with a tegfr safety switch exhibit potent toxicity control. Blood Cancer J. 14, 98. 10.1038/s41408-024-01082-y 38890292 PMC11189405

[B26] Lonez C. Breman E. (2024). Allogeneic car-t therapy technologies: has the promise been met? Cells 13, 146. 10.3390/cells13020146 38247837 PMC10814647

[B27] Longhurst H. Fijen L. Lindsay K. Butler J. Golden A. Maag D. (2022). *In vivo* crispr/cas9 editing of klkb1 in patients with hereditary angioedema: a first-in-human study. Ann. Allergy, Asthma and Immunol. 129, S10–S11. 10.1016/j.anai.2022.08.536

[B28] McGuirk J. P. Tam C. S. Kröger N. Riedell P. A. Murthy H. S. Ho P. J. (2022). Ctx110 allogeneic crispr-cas9-engineered car t cells in patients (pts) with relapsed or refractory (r/r) large b-cell lymphoma (lbcl): results from the phase 1 dose escalation carbon study. Blood 140, 10303–10306. 10.1182/blood-2022-166432

[B29] Moradi V. Khodabandehloo E. Alidadi M. Omidkhoda A. Ahmadbeigi N. (2024). Progress and pitfalls of gene editing technology in car-t cell therapy: a state-of-the-art review. Front. Oncol. 14, 1388475. 10.3389/fonc.2024.1388475 38912057 PMC11190338

[B30] Munari E. Mariotti F. R. Quatrini L. Bertoglio P. Tumino N. Vacca P. (2021). Pd-1/pd-l1 in cancer: pathophysiological, diagnostic and therapeutic aspects. Int. Journal Molecular Sciences 22, 5123. 10.3390/ijms22105123 34066087 PMC8151504

[B31] Musunuru K. Grandinette S. A. Wang X. Hudson T. R. Briseno K. Berry A. M. (2025). Patient-specific *in vivo* gene editing to treat a rare genetic disease. N. Engl. J. Med. 392, 2235–2243. 10.1056/NEJMoa2504747 40373211 PMC12713542

[B32] Narisawa-Saito M. Kiyono T. (2007). Basic mechanisms of high-risk human papillomavirus-induced carcinogenesis: roles of e6 and e7 proteins. Cancer Science 98, 1505–1511. 10.1111/j.1349-7006.2007.00546.x 17645777 PMC11158331

[B33] Nathans J. Hogness D. S. (1984). Isolation and nucleotide sequence of the gene encoding human rhodopsin. Proc. Natl. Acad. Sci. 81, 4851–4855. 10.1073/pnas.81.15.4851 6589631 PMC391589

[B34] National Institutes of Health (2022). The basics. Available online at: https://www.nih.gov/health-information/nih-clinical-research-trials-you/basics.

[B35] National Library of Medicine (2025). Rho gene: medlineplus genetics. Available online at: https://medlineplus.gov/genetics/gene/rho/#conditions.

[B36] Oppermann M. (2004). Chemokine receptor ccr5: insights into structure, function, and regulation. Cell. Signalling 16, 1201–1210. 10.1016/j.cellsig.2004.04.007 15337520

[B37] Ottaviano G. Georgiadis C. Gkazi S. A. Syed F. Zhan H. Etuk A. (2022). Phase 1 clinical trial of crispr-engineered car19 universal t cells for treatment of children with refractory b cell leukemia. Sci. Translational Medicine 14, eabq3010. 10.1126/scitranslmed.abq3010 36288281

[B38] Ozdemirli M. Loughney T. M. Deniz E. Chahine J. J. Albitar M. Pittaluga S. (2024). Indolent cd4+ car t-cell lymphoma after cilta-cel car t-cell therapy. N. Engl. J. Med. 390, 2074–2082. 10.1056/NEJMoa2401530 38865661

[B39] Quigley H. A. Hohman R. M. Addicks E. M. Massof R. W. Green W. R. (1983). Morphologic changes in the Lamina cribrosa correlated with neural loss in open-angle glaucoma. Am. Journal Ophthalmology 95, 673–691. 10.1016/0002-9394(83)90389-6 6846459

[B40] Rinaldi I. Muthalib A. Edina B. C. Wiyono L. Winston K. (2022). Role of anti-b-cell maturation antigen (bcma) in the management of multiple myeloma. Cancers 14, 3507. 10.3390/cancers14143507 35884566 PMC9317279

[B41] Rostami N. Gomari M. M. Choupani E. Abkhiz S. Fadaie M. Eslami S. S. (2024). Exploring advanced crispr delivery technologies for therapeutic genome editing. Small Sci. 4, 2400192. 10.1002/smsc.202400192 40212235 PMC11935293

[B42] Ruberg F. L. Berk J. L. (2012). Transthyretin (ttr) cardiac amyloidosis. Circulation 126, 1286–1300. 10.1161/CIRCULATIONAHA.111.078915 22949539 PMC3501197

[B43] Rueda J. de Miguel Beriain Í. Montoliu L. (2024). Affordable pricing of crispr treatments is a pressing ethical imperative. CRISPR Journal 7, 220–226. 10.1089/crispr.2024.0042 39392045

[B44] Sasaki T. Hiroki K. Yamashita Y. (2013). The role of epidermal growth factor receptor in cancer metastasis and microenvironment. BioMed Research International 2013, 546318. 10.1155/2013/546318 23986907 PMC3748428

[B45] Scialo F. Daniele A. Amato F. Pastore L. Matera M. G. Cazzola M. (2020). Ace2: the major cell entry receptor for sars-cov-2. Lung 198, 867–877. 10.1007/s00408-020-00408-4 33170317 PMC7653219

[B46] Singh A. Irfan H. Fatima E. Nazir Z. Verma A. Akilimali A. (2024). Revolutionary breakthrough: Fda approves casgevy^TM^, the first crispr/cas9 gene therapy for sickle cell disease. Ann. Med. Surg. 86, 10–1097. 10.1097/MS9.0000000000002146 PMC11305803 39118728

[B47] Terrett J. Kalaitzidis D. Dequeant M. Karnik S. Ghonime M. Guo C. (2023). Ctx112 and ctx131: next-generation crispr/cas9-engineered allogeneic (allo) car t cells incorporating novel edits that increase potency and efficacy in the treatment of lymphoid and solid tumors. Cancer Res. 83, 7–Suppl. 10.1158/1538-7445.AM2023-ND02

[B48] Vertex Pharmaceuticals (2024). Vertex announces US FDA Approval of CASGEVY^TM^ (exagamglogene autotemcel) for the Treatment of Transfusion-Dependent Beta Thalassemia. Available online at: https://investors.vrtx.com/news-releases/news-release-details/vertex-announces-us-fda-approval-casgevytm-exagamglogene.

[B49] Wang Z. Chen M. Zhang Y. Liu Y. Yang Q. Nie J. (2020). Phase i study of crispr-engineered car-t cells with pd-1 inactivation in treating mesothelin-positive solid tumors. J. Clin. Oncol. 38, 3038. 10.1200/JCO.2020.38.15_suppl.3038

[B50] Wei A. Yin D. Zhai Z. Ling S. Le H. Tian L. (2023). *In vivo* crispr gene editing in patients with herpetic stromal keratitis. Mol. Ther. 31, 3163–3175. 10.1016/j.ymthe.2023.08.021 37658603 PMC10638052

[B51] Witkowsky L. Norstad M. Glynn A. R. Kliegman M. (2023). Towards affordable crispr genomic therapies: a task force convened by the innovative genomics institute. Gene Therapy 30, 747–752. 10.1038/s41434-023-00392-3 37935852 PMC10678297

[B52] Xiao X. Huang S. Chen S. Wang Y. Sun Q. Xu X. (2021). Mechanisms of cytokine release syndrome and neurotoxicity of car t-cell therapy and associated prevention and management strategies. J. Exp. and Clin. Cancer Res. 40, 367. 10.1186/s13046-021-02148-6 34794490 PMC8600921

[B53] Xu L. Wang J. Liu Y. Xie L. Su B. Mou D. (2019). Crispr-edited stem cells in a patient with hiv and acute lymphocytic leukemia. N. Engl. J. Med. 381, 1240–1247. 10.1056/NEJMoa1817426 31509667

[B54] Yoshizumi I. Krupovic M. Forterre P. (2018). History of crispr-cas from encounter with a mysterious repeated sequence to genome editing technology. J. Bacteriology 200, 10–1128. 10.1128/JB.00580-17 PMC5847661 29358495

[B55] Zetsche B. Gootenberg J. S. Abudayyeh O. O. Slaymaker I. M. Makarova K. S. Essletzbichler P. (2015). Cpf1 is a single rna-guided endonuclease of a class 2 crispr-cas system. Cell 163, 759–771. 10.1016/j.cell.2015.09.038 26422227 PMC4638220

[B56] Zhang C. Liu J. Zhong J. F. Zhang X. (2017). Engineering car-t cells. Biomark. Research 5, 22. 10.1186/s40364-017-0102-y 28652918 PMC5482931

